# Report of a case of giant rhinoscleroma: CT and MRI

**DOI:** 10.1259/bjrcr.20180027

**Published:** 2018-07-10

**Authors:** Dalia Ibrahim, Ahmed Fayed

**Affiliations:** 1 Department of Radiology, Kasr Elainy, Faculty of medicine, Cairo university, Giza, Egypt

## Abstract

Rhinoscleroma is a chronic granulomatous bacterial infection caused by the gram-negative bacillus *Klebsiella rhinoscleromatis*. It predominately involves the nasal cavities but it can also involve the rest of the upper respiratory tract. Hypertrophic stage of rhinoscleroma may cause large tumor masses which could mimic neoplasm. Radiological imaging is essential for differentiation of rhinoscleroma from other granulomatous and malignant lesions. Imaging is also an important tool for detection of disease extensions and follow up post therapy. We illustrate the radiographic features of a patient with a giant rhinoscleroma using CT, MRI and CT virtual bronchoscopy for prompt diagnosis, assessment of disease extensions before therapy and follow up after therapy. The diagnosis was confirmed by tissue biopsy and culture. The patient received medical antibiotic treatment for 3 months after surgical excision of the lesion.

A 37-year-old male who had lived in a rural region in Egypt presented with large nasal masses and epistaxis. The disease had appeared 15 years before; it started with non-obstructing nasal polyps which indicated surgical excision. The histological and bacteriological examinations revealed rhinoscleroma. The patient subsequently received antibiotic treatment. The nasal polyps persisted to recur for 14 times for which he required surgical excision and antibiotic treatment each time. The patient did not receive any medical or surgical treatment for the last 2 years; after which he presented to our facility with large nasal and palatal masses. Physical examination revealed large nasal and palatal erythematous soft tissue masses which bleed on contact (Figure 1).

**Figure 1.**
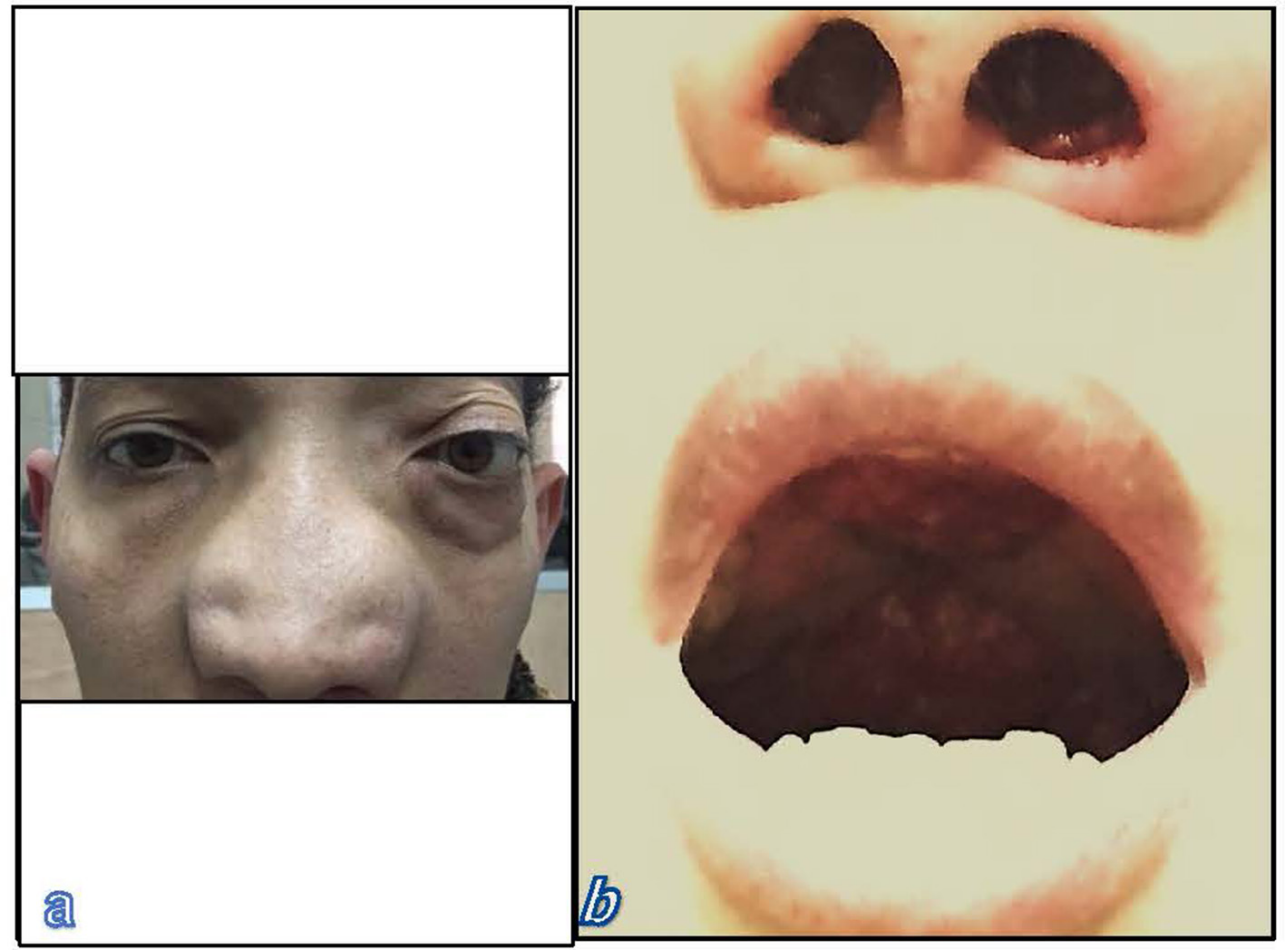
Clinical photographs of a patient with rhinoscleroma. Photograph (a) demonstrates nasal enlargement and proptosis. Photograph (b) shows reddish granular masses within nasal cavities and hard palate.

## Investigations

**Figure 2.**
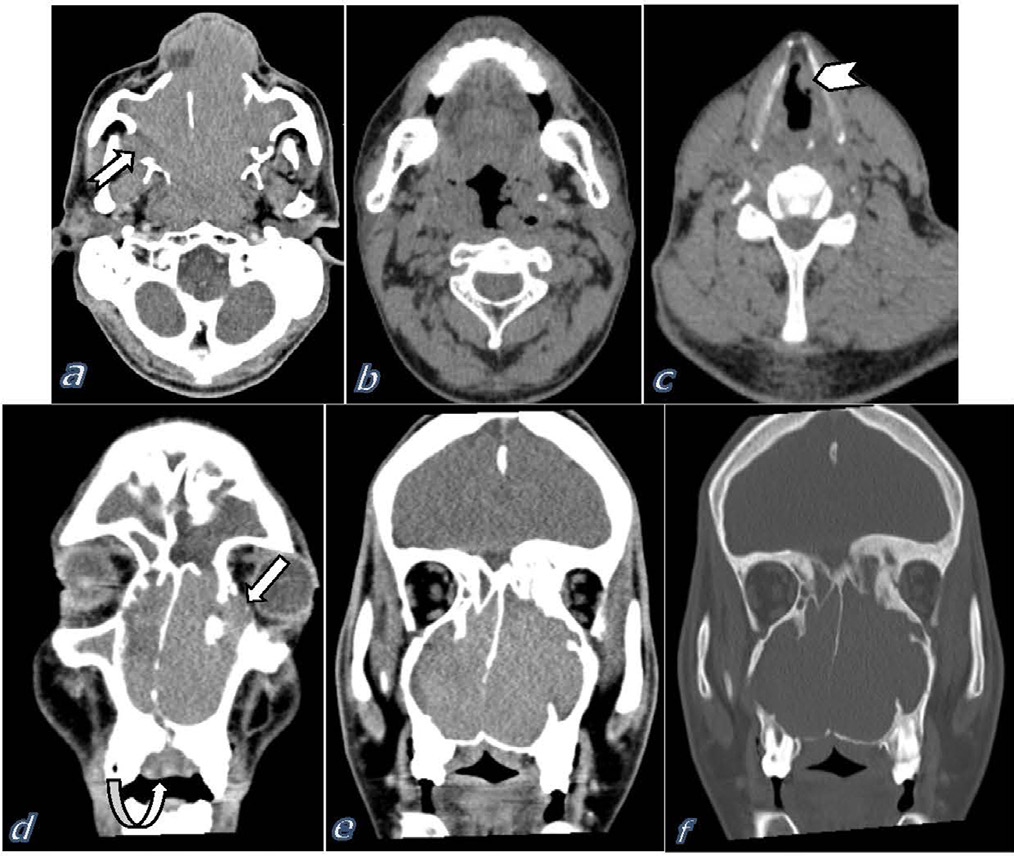
Contrast-enhanced CT scan of the paranasal sinuses, (a) axial image showed large soft tissue masses within the nasal cavities extended laterally into the maxillary antra and posteriorly into the nasopharynx. The masses show well-defined margins and homogenous high attenuation values. Bone absorption of the right lateral maxillary antrum wall is noted with small extramaxillary sinus extension (notched arrow), (b) axial image showed circumferential oropharyngeal mural thickening and lumen narrowing, (c) axial image of the neck showed laryngeal affection with nodular thickening of the left false vocal cord (chevron), (d) coronal image of the paranasal sinuses showed large masses within nasal fossae with small extension into the left orbital cavity (block arrow), also note the hard palatal mass (curved arrow), (e) coronal image of the paranasal sinuses showed large masses within nasal fossae, both maxillary antra and ethmoidal air cells, (f) coronal image bone window showed areas of bone sclerosis and areas of bone thinning (pressure atrophy).

Contrast-enhanced CT scan of the paranasal sinuses revealed large non-enhancing soft tissue masses of high attenuation values within the nasal cavities which extended into both maxillary antra and ethmoidal air cells. The masses extended posteriorly through the posterior choana into the nasopharynx. It caused obstruction of the cartilaginous portions of the Eustachian tubes with subsequently retained secretions in both middle ear cavities. The masses had also occluded the draining ostia of the paranasal sinuses that resulted in the accumulation of retained secretions within the related sinuses and the formation of large left sphenoid mucocele. The left nasal mass extended into the left orbital cavity with subsequent left eye proptosis. The adjacent bony boundaries appeared sclerotic with areas of bone thinning (pressure atrophy) and bone absorption. Bone absorption was seen on the right lateral maxillary sinus wall with small extramaxillary sinus extension. Oropharynx showed lumen narrowing and concentric irregular mural thickening with multiple hard and soft palatal masses. Laryngeal involvement was mainly supraglottic in the form of nodular thickening of the aryepiglottic folds and false vocal cords. No subglottic involvement (Figure 2).

**Figure 3.**
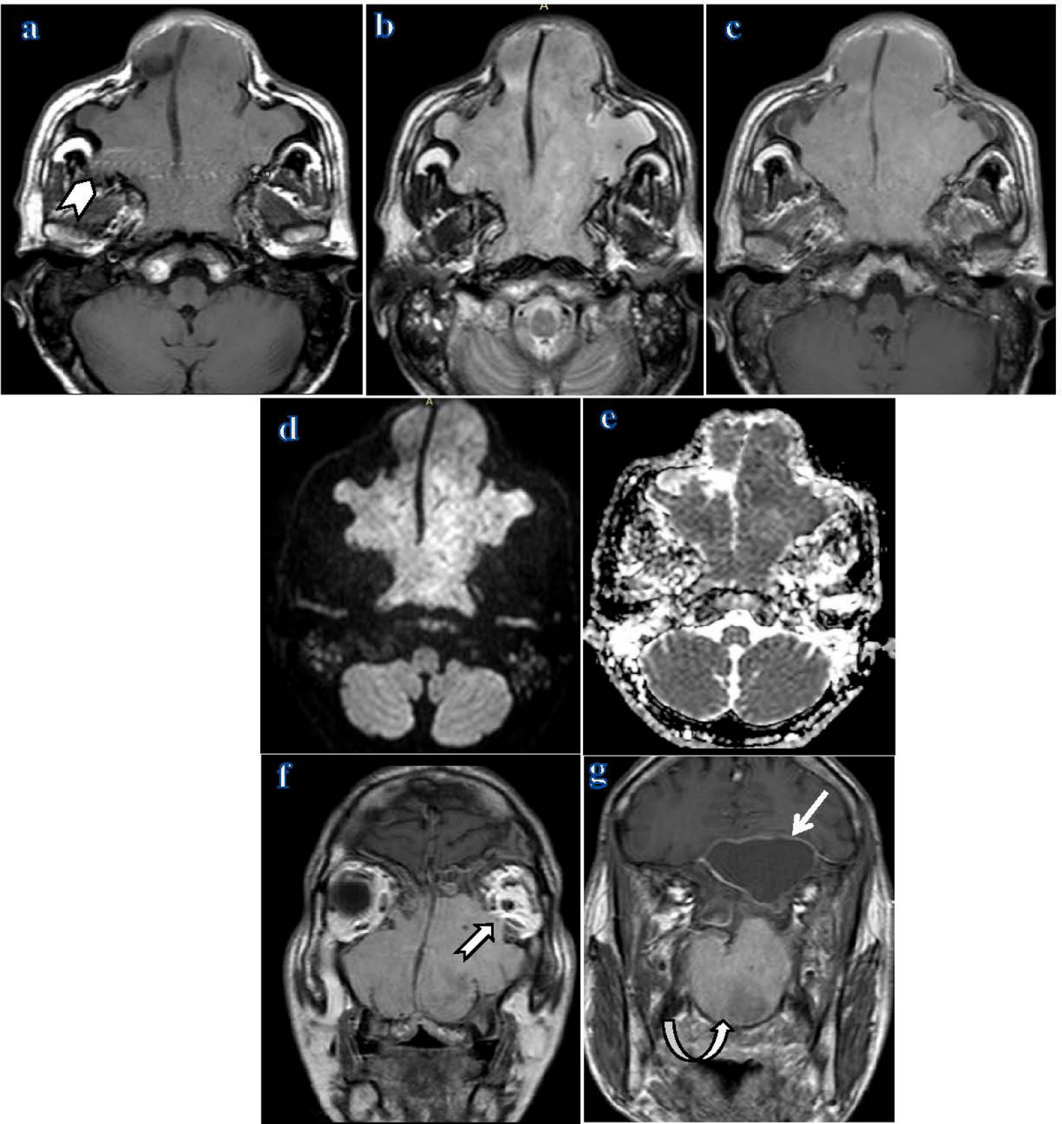
Contrast-enhanced MRI examination of the paranasal sinuses; (a) The axial *T*_1_ weighted image showed bilateral large masses of intermediate signal intensity within nasal cavities, nasopharynx, both maxillary antra, with small right extramaxillary sinus extension (chevron), (b) Axial *T*_2_ weighted image showed diffuse high signal intensity of the masses, (c) axial *T*_1_ weighted image post-gadolinium contrast administration showed diffuse homogeneous post-contrast enhancement. (d) Axial DWI showed diffusion restriction of the masses, (e) axial ADC map showed low attenuation values, (f) coronal *T*_1_ weighted image post-gadolinium contrast administration showed homogeneously enhancing masses within nasal cavities, both maxillary antra and ethmoidal air cells with left intraorbital extension (notched arrow), (g) coronal *T*_1_ weighted image post-gadolinium contrast administration showed the enhancing mass within the nasopharynx (curved arrow) and the large left sphenoid mucocele which showed marginal post contrast enhancement (straight arrow). ADC, apparent diffusion co-efficient; DWI, diffusion weighted imaging.

MRI complemented the CT findings and confirmed the tumoral appearance of this mass with better delineation of the mass extensions. It was helpful for differentiation of the mass from the adjacent retained secretions and sphenoid mucocele by their signal intensity characteristics. The lesion elicits intermediate signal on *T*_1_ and high signal on *T*_2_ weighted images. It showed diffusion restriction and homogenous post contrast enhancement (Figure 3).

**Figure 4.**
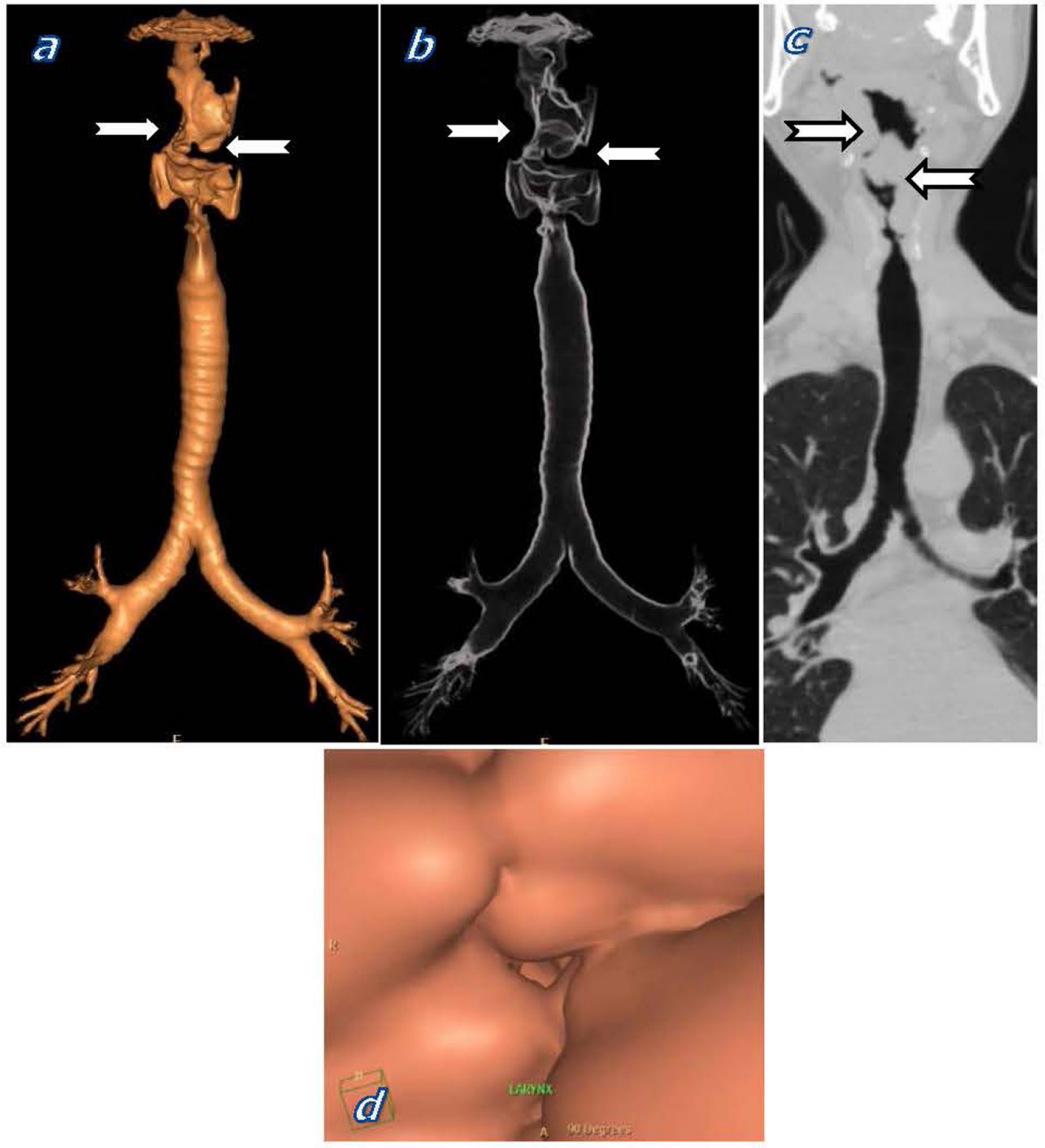
Volume-rendered 3D CT virtual bronchoscopy, (b) 3D transparency view, (c) coronal MPR, and (d) endoluminal view showed laryngeal lumen narrowing and irregular nodular thickening of the laryngeal wall (notched arrows). Normal appearance of the tracheobronchial tree. 3D, three-dimensional; MPR, multiplanar reconstruction.

CT virtual bronchoscopy revealed supraglottic laryngeal involvement which is not the typical site of laryngeal involvement of scleroma. Scleroma usually affects the subglottic region. CT showed supraglottic laryngeal lumen narrowing and diffuse nodular thickening of the aryepiglottic folds and false vocal cords (Figure 4). The tracheobronchial tree showed normal appearance with no evidence of wall thickening or lumen narrowing.

**Figure 5.**
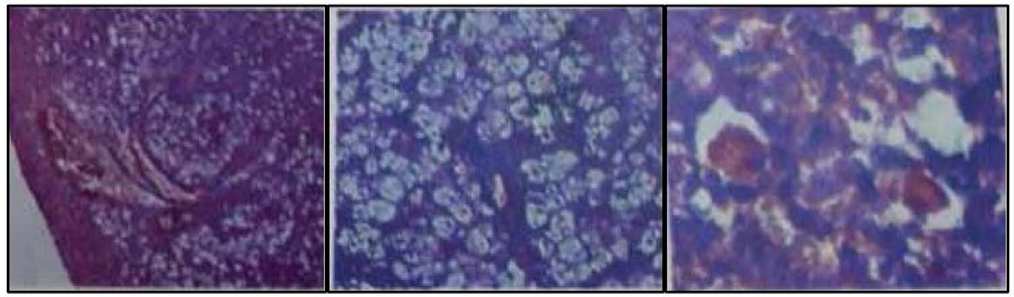
Photomicrographs show sheets of inflammatory cell infiltrates and bands of fibrosis. Inflammatory cells mostly formed of large vacuolated histiocytes (Mikulicz cells) and plasma cells.

Histopathological examination of the nasal masses revealed pieces of tissue showing stretched stratified squamous covering with focal ulceration overlying dense inflammatory reaction mostly formed of lymphocytes, plasma cells with Russell bodies, together with foamy macrophages (Mikulicz’s cells) showing abundant clear vacuolated cytoplasm and intracytoplasmic bacilli (Figure 5).

## Treatment and follow up

The patient had surgical excision of the nasal masses followed by antibiotic treatment in the form rifampicin treatment 150 mg twice daily for 4 months. Follow up CT scan following surgical excision and antibiotic treatment showed significant improvement with absent residual or recurrent nasal masses.

## Discussion

Rhinoscleroma is a chronic granulomatous bacterial disease of the nose that can sometimes infect the upper respiratory tract.^1^ It is caused by gram-negative bacillus *Klebsiella* *rhinoscleromatis*.^1 ^Rhinoscleroma predominantly involves the nasal mucosa, but it can also involve the larynx; nasopharynx; oral cavity; paranasal sinuses; trachea, bronchi or soft tissues of the lips and nose.^2^ Our patient had large extensive masses with the involvement of multiple sites that included the nose, nasopharynx, paranasal sinuses, left orbit, oropharynx, and larynx.

 The disease is endemic in Egypt and many other African countries, South-East Asia, Mexico, Central and South America, and Eastern parts of Europe. However, the incidence of rhinoscleroma may increase with current trends in migration.^3^ Infection with rhinoscleroma is usually associated with low socioeconomic status, poor hygiene, and prolonged contact with infected individuals.^4^

 Rhinoscleroma is usually classified clinically and pathologically into the following three stages: the catarrhal (or atrophic) stage, the proliferative (or granulomatous) stage, and the fibrotic (or sclerotic) stage.^1^

 Radiological imaging is essential for early diagnosis of rhinoscleroma, differentiation of rhinoscleroma from similar granulomatous and neoplastic lesions, and detection of disease extensions and complications.^1^ Imaging is also important for the determination of treatment and follow up post-therapy.^1^ CT and MRI are the recommended radiological examinations. On CT scan, bilateral or unilateral expanded nasal masses of variable size.^1^ Characteristic lesions are homogeneous, hyperdense and non-enhancing masses with well-defined borders.^3^ Scleroma usually extends into the maxillary sinuses through the ostiomeatal units.^5^ Scleroma can also extend into the ethmoid and sphenoid sinus with intracranial extension.^5^ It lines the sinus wall causing bone sclerosis, bone thinning (pressure atrophy) or it may absorb it with extramaxillary sinus extension. No bone destruction which helps to differentiate it from malignant lesions.^1^ Nasal masses can also obstruct the ostiomeatal units, and secretions may be retained in the related sinuses. CT demonstrates

 Extension of rhinoscleroma to the pharynx has a variable incidence which varies from 18 to 43% in different areas.^6^Pharyngeal scleroma causes nodular infiltration of the oropharynx, tonsillar fossa, soft and hard palate.^7^ The incidence of laryngotracheal involvement varies from 15 to 80%. In scleroma, the usual site of laryngeal involvement is the glottic–subglottic region.^8^ It shows concentric narrowing of the airway, thickening of the tracheal wall and nodular thickening of the tracheal mucosa.^8^

 On MRI, the hypertrophic stage of rhinoscleroma has characteristic mild to marked high signal intensity on both *T*_1_ and *T*_2_ weighted images. That characteristic high signal intensity is secondary to increased protein content within Mikulicz cells and Russell bodies.^4^ On *T*_2_ weighted images, it appears hyperintense due to its high cellular component; this is usually associated with hypointense foci of fibrosis. The signal of the lesion changes with time secondary to increase of macromolecular protein concentration and a decrease of the amount of free water, that results in a reduction of *T*_2_ and *T*_1_ relaxation times.^1^ The lesion typically shows an inhomogeneous pattern of contrast enhancement following contrast administration secondary to the presence of areas of fibrosis.^4^ At diffusion MRI, the lesion shows diffusion restriction with low apparent diffusion coefficients values which could mimic malignancy secondary to high cellularity and the presence of fatty cells within the lesion.^9, 10^

### 

 The differential diagnosis includes granulomatous diseases which can mimic rhinoscleroma, infectious granulomatous processes: bacterial (tuberculosis, actinomycosis, leprosy, and syphilis), fungal (histoplasmosis, sporotrichosis, and paracoccidioidomycosis) and parasitic (mucocutaneous leishmaniasis) or noninfectious granulomatous diseases (granulomatosis with polyangiitis and sarcoidosis), and neoplasms (lymphomas and carcinomas).^11, 12^ Expansion of the nasal cavities, scalloping of the sinus wall, lack of bone destruction and hyperintensity of the nasal mass on *T*_1_ weighted images help to differentiate rhinoscleroma from nasal malignancies.^1^

 Diagnosis is made based on pathology and bacteriology. Histopathology shows an infiltrate of chronic inflammatory cells, the hallmark of rhinoscleroma is the presence of “Mikulicz cells” (foamy macrophages) and “Russell bodies” (reddish elliptical structures bigger than plasma cells likely representing degenerated plasma cells).^4^ A specific diagnosis is made by identification of rod-shaped bacilli that are positive to periodic acid-Schiff and Warthin-Starry stain.^13^

 To avoid disease recurrence and complications, it is essential to diagnose the disease early and to use prolonged therapy. Neglected untreated patients tend to progress to involve other parts of the respiratory tract. Disease extension to the trachea can lead to progressive airway obstruction.^14^

 Treatment of rhinoscleroma is antibiotic therapy.^1^ Surgical intervention is usually reserved for excision of obstructing masses or tracheostomy in case of airway obstruction.^1^

## Learning points

1. Rhinoscleroma is a chronic granulomatous bacterial infection of the nose and upper respiratory tract.
2. Rhinoscleroma should be considered in the differential diagnosis of granulomatous diseases of the nose in endemic areas.
3. Hypertrophic stage of rhinoscleroma can lead to the formation of large masses which could mimic neoplasm.
4. Imaging is essential for the diagnosis of rhinoscleroma and for differentiation of rhinoscleroma from other granulomatous and malignant nasal lesions.
5. Imaging is also helpful for determination of therapy and follow up post therapy.
